# Comparison of Sensory and Electronic Tongue Analysis Combined with HS-SPME-GC-MS in the Evaluation of Skim Milk Processed with Different Preheating Treatments

**DOI:** 10.3390/molecules24091650

**Published:** 2019-04-26

**Authors:** Minghui Pan, Lingjun Tong, Xuelu Chi, Nasi Ai, Yungang Cao, Baoguo Sun

**Affiliations:** 1 Beijing Advanced Innovation Center for Food Nutrition and Human Health, Beijing Engineering and Technology Research Center of Food Additives, Beijing Higher Institution Engineering Research Center of Food Additives and Ingredients, Beijing Technology & Business University, Beijing 100048, China; 1830202061@st.btbu.edu.cn (M.P.); m18754030882@163.com (L.T.); chi_xl@163.com (X.C.); sunbg@btbu.edu.cn (B.S.); 2 School of Food and Biological Engineering, Shanxi University of Science & Technology, Xi′an 710021, China; caoyungang@sust.edu.cn

**Keywords:** skim milk, sensory, volatile compounds, preheat treatment, PCA

## Abstract

It is well known that the flavor of skim milk is inferior to whole milk due to the lack of fat. With the popularity of low-fat dairy products, improving the flavor of skim milk is a main focus for food scientists. During the production of skim milk, preheating treatments have a significant effect for the flavor of skim milk. In this study, to explore the optimal processing conditions, milk was preheated at 30 °C, 40 °C, 50 °C, 60 °C for 30 min prior to defatting. When the optimal temperature was determined, milk was then preheated at the optimal temperature for 10 min, 20 min, 30 min, 40 min and 50 min, respectively, to obtain the best preheating time. Distinctions between skim milk samples with different processing conditions were studied by sensory evaluation, e-tongue and HS-SPME-GC-MS analysis. Principle components analysis (PCA) and cluster analysis (CA) were selected to associate with e-tongue results and compare the similarities and differences among the skim milks. Sensory and e-tongue results matched and both showed that a preheating temperature of 50 °C and 30 min time might be the optimal combination of processing conditions. Thirteen volatiles, including ketones, acids, aldehydes, alcohols, alkanes and sulfur compounds, were analyzed to evaluate flavor of the skim milks produced by different preheating treatments. Combined with previous studies, the results indicated that most volatile compounds were decreased by reducing the fat concentration and the typical compound 2-heptanone was not detected in our skim milk samples.

## 1. Introduction

Cow milk has a recognized role in human diet reflected in its inclusion in the most recognized international health organization′s dietary recommendation model [1]. There is some consensus that milk is on average composed of 87% water, 4–5% lactose, 3% protein, 3–4% fat, 0.8% minerals, and 0.1% vitamins [2]. Meanwhile, determined by the contents of milk fat, cow milk is classified as whole milk and skim milk [3]. The nutritional value of cow milk is evaluated by its nutritional richness in high biological-value proteins, several B complex vitamins and minerals such as calcium [4,5], while milk fat is mainly in charge of the flavor and sensory feel and unique in making a direct contribution to sensory aroma and flavor perception [6,7,8,9]. It is reported that fat is not only responsible for many of milk’s sensory attributes and flavor, but also affects appearance, texture, and palatability [10,11] Several studies have carried out that sensory properties of milk vary with fat content, and it is clear that milk fat plays a role in appearance and texture attributes of fluid milk [12,13,14,15,16]. However, studies have shown that a high fat diet increases the fat in serum and liver, which causes intestinal dysfunction, intestinal barrier function damage and an increase of circulating lipopolysaccharide [17]. Besides, dietary recommendations began changing in the 1980s, and the reason was based on a research linking fat consumption to cardiovascular disease and obesity [18]. Whole milk consumption has shown a declining trend since 1970, and more consumers have shifted from whole milk to skim milk [19]. Therefore, a low-fat diet became an overarching ideology, promoted by physicians, governments and food industries, but the flavor of skim milk is inferior compared to whole milk. Flavor changes caused by the lack of fat are the reason why many general consumers are still unwilling to accept skim milk. Hence, improving the flavor of skim milk is a main focus of food scientists.

The flavor of skim milk should be improved by processing but not by adding ingredients. New and effective methods of whole milk processing have been developed, including high pressure [20], ultrasound [21] and preheating treatments [22]. The advantages and disadvantages of each of these methods are listed in Table 1. Preheating treatment is always a necessary step in the processing of dairy products, and its different intensity has significant effects on the milk, such as heat-induced denaturation of milk protein [23,24], cross-linking of whey protein and casein [25], fat oxidation [26], deterioration of flavor at ultrahigh temperature [27]. For this reason, we attempted to preheat at a slight temperature softly prior to defatting to obtain a favorable impact on the flavor of skim milk.

Various methods have been popularized for food analysis, including milk, such as gas chromatography-mass spectrometry (GC-MS), electronic tongue (e-tongue) and electronic nose (e-nose). E-nose are a focus of applications within the food quality monitoring field [30], especially in the detection of adulteration of edible oils [31], and food classification based on shelf-life [32,33]. The operating principles and configuration of e-nose have been reported by previous studies [34,35].

The aim of this study was to evaluate the flavor and taste changes after skim milk processing by preheating at different temperatures and for different times prior to defatting. For this purpose, a sensory evaluation analysis was performed on samples subjected to different treatments, followed by e-tongue analysis supplemented with principal component analysis (PCA) and cluster analysis (CA). The volatile components were isolated by headspace solid-phase micro-extraction (HS-SPME, PDMS/DVB fiber) and further analyzed by GC-MS.

## 2. Results

### 2.1. Sensory Evaluation Analysis

**Figure 1:**
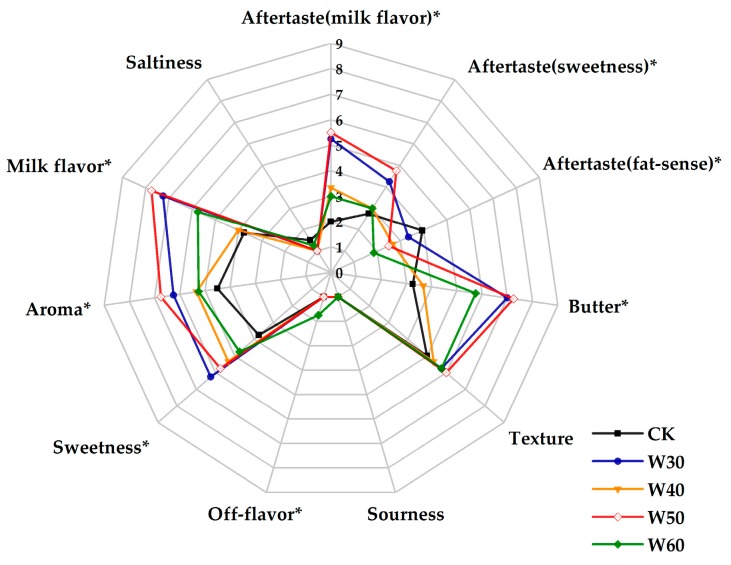
Sensory evaluation of the skim milk samples preheated at different temperature.

**Figure 2:**
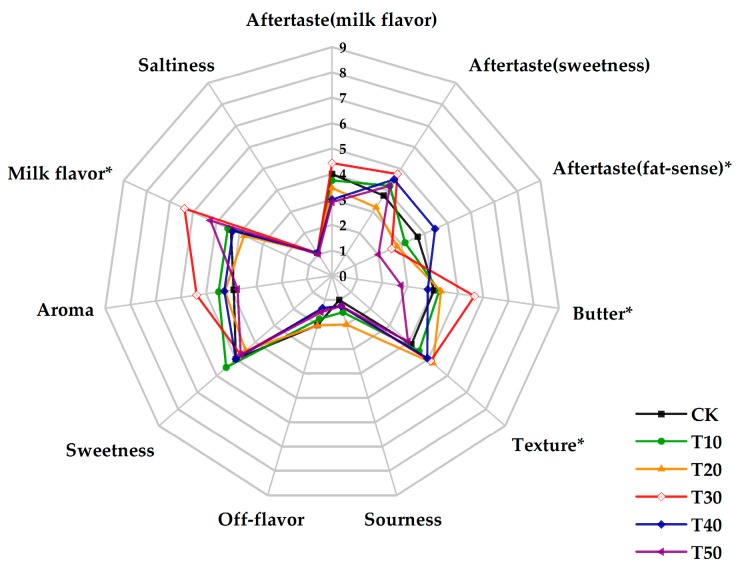
Sensory evaluation of the skim milk samples preheated for different time.

The number of determinations for sensory evaluation in the skim milks processed with different treatments are shown in Figure 1 and Figure 2 with aftertaste (milk flavor, sweetness and fat-sense), texture, butter, milk flavor, saltiness, sourness, off-flavor, sweetness and aroma.

Sensory scores among samples produced by processing with different temperature are shown in Figure 1. There were no significant differences in “sourness”, “saltiness”, “texture” among samples. Sample W50 showed significantly (*p* < 0.05) higher scores for “aftertaste (milk flavor)”, “aftertaste (sweetness)”, “milk flavor” and “butter” relative to the control group (CK), W40 and W60. In addition, W50 held significantly (*p* < 0.05) a higher score in “aroma” and “sweetness” and a lower score in “aftertaste (fat-sense)” compared to CK. W60 held a significant (*p* < 0.05) higher score in “off-flavor” compared to the others.

Sensory scores among samples produced by preheating for different time are shown in Figure 2. No significant differences in “aftertaste (milk flavor)”, “aftertaste (sweetness)”, “aroma”, “sweetness”, “off-flavor”, “sourness”, “saltiness” occurred among the samples. T30 held significantly (*p* < 0.05) higher scores in “milk flavor” compared to CK, T10, T20 and T50, “texture” compared to T50 and “butter” relative to the others. The score for “aftertaste (fat-sense)” of T40 was higher significantly (*p* < 0.05) than T30 and T50.

“Milk flavor”, “butter”, “aroma” and “off-flavor” are several important indicators when evaluating a new dairy product. From Figure 1 and Figure 2, samples produced by preheating at 50 °C and for 30 min are the optimal processing conditions due to the higher scores in “milk flavor”, “butter” and “aroma”, and a lower score in “off-flavor” relative to other processing conditions.

### 2.2. Analysis with e-Tongue Using PCA and CA

All milk samples W30, W40, W50, W60, T10, T20, T30, T40, T50, the control group (CK) and raw milk (RM) were analyzed by the e-tongue. In order to diagnose and characterize the correlation among the taste of samples produced by processing different treatments, PCA and CA were mapped using the relative voltage response of the e-tongue sensors to milk samples. The subsequent PCA of samples processing with different preheating temperature and time have 96%, 93% of the variations represented by the first two principal components. The variations seem to provide enough grouping information on the samples [36].

**Figure 3:**
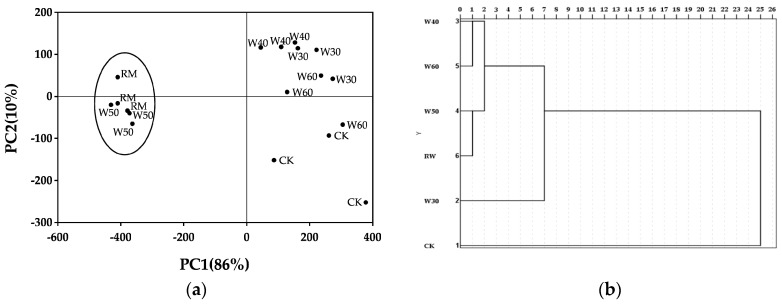
Classification of all skim milk samples preheated at different temperature: (**a**) Score plot after PCA of the samples. (**b**) Dendrogram from CA of the samples.

The PCA score plot of samples produced by preheating at different temperature is shown in Figure 3a and the dendrogram of the samples is shown in Figure 3b. The PCA and CA classifications coincide. RM and W50 are classified as the same cluster, which indicated that the taste of W50 was the most similar to RM. There are also great similarities between W30, W40 and W60. Meanwhile, the results of PCA (Figure 3a), CA (Figure 3b) and sensory analysis (Figure 1) were consistent and fully demonstrate taste of sample preheated at 50 °C was preferred.

**Figure 4:**
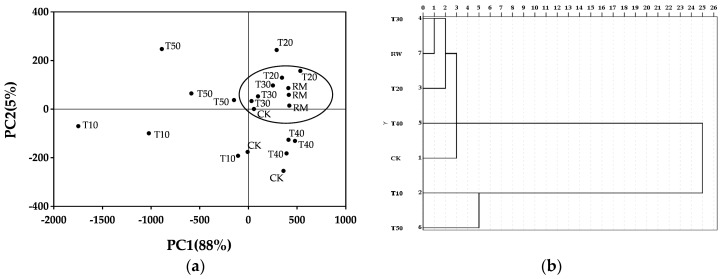
Classification of all skim milk samples preheated for different time: (**a**) Score plot after PCA of the samples. (**b**) Dendrogram from CA of the samples.

The classifications of samples produced by preheating for different time are shown in Figure 4. From Figure 4a, seven different samples were clearly dispersed. T30 was classified as a same cluster with RM. In addition, parts of CK and T20 was classified as the same group with RM and T30. This result indicated that the similarity of taste between T30 and RM was highest and they held slight similarity with T20 and CK. Other groups held distinct differences on the taste.

Meanwhile, combined with sensory evaluation, the taste of preheating for 30 min was perfected. Therefore, combining the results of the sensory evaluation with PCA and CA, preheating at 50 °C for 30 min before the defatting processing was significantly (*p* < 0.05) conducive to improving the taste of skim milk relative to other processing conditions.

### 2.3. GC-MS Analysis

**Figure 5:**
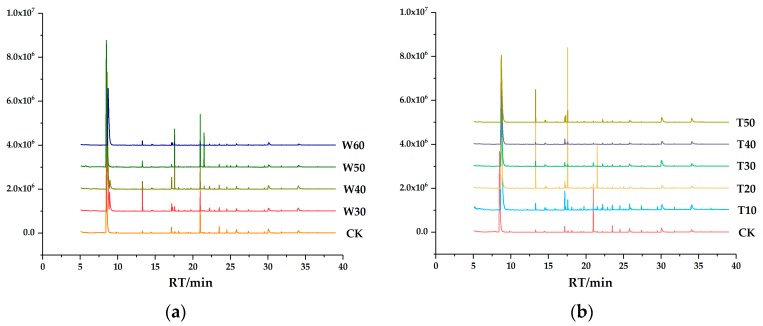
Comparison of TIC profile of the volatiles from the preprocessing skim milk samples. (**a**) The TIC profile of the volatiles from the skim milk samples produced by preheating at different temperature. (**b**) The TIC profile of the volatiles from the skim milk samples produced by preheating for different time.

Solid-phase micro-extraction (SPME) is optimized to discriminate aroma release from the different milk compared to static headspace and dynamic headspace [37]. The flavor of milk is influenced by processing, season, cows’ growing environment and calving number. Volatile compounds were isolated from samples W30, W40, W50, W60, T10, T20, T30, T40, T50 and CK using SPME-GC-MS. Comparison of typical total ion currency (TIC) profile of the volatiles from the preprocessing skim milk samples were shown in Figure 5. Thirteen major compounds was analyzed, including acids, alcohols, aldehydes, ketones, alkanes, and sulfur compounds, and the volatile compounds are shown in Table 2 and Table 3.

From Table 2, there are no significant differences in the content of total ketones and alcohols between samples with different preheating temperatures. W50 displayed a significant (*p* < 0.05) high content in total acids compared to W30, W40, W60 and slightly higher than CK. The total aldehydes of W50 were significantly (*p* < 0.05) more abundant compared to W40, W60 and CK, while the differences between W50 and W30 was not significant. Tetradecane was produced when preheating at 30 °C, 50 °C and 60 °C and the difference between samples was significant (*p* < 0.05). The contents of sulfur compound (dimethyl sulfone) in CK and W50 were 0.10 ± 0.00 μg/L and 0.15 ± 0.10 μg/L, respectively.

Major volatile compounds of the samples preheated for different times are shown in Table 3. There was no significant difference in the content of total alcohols. Content of total ketones in T30 was significantly (*p* < 0.05) lower compared to T20, but without significant differences from the other samples. Meanwhile, T30 held the highest content of total acids, while the content of total aldehydes was near to T10, T20, T40 and CK and significantly (*p* < 0.05) lower than T50. Tetradecane was produced by all samples after preheating for different times, while CK and T20 displayed a significantly (*p* < 0.05) higher content compared to other groups. Except for the CK and T30 samples, no sulfur compounds were produced.

## 3. Discussion

### 3.1. Sensory Evaluation Analysis

During preheating treatment of milk, various reactions take place, including denaturation and aggregation of whey protein, formation of complexes between whey proteins, caseins and fat globules which might cause the lack of flavor after milk defatting. However, studies have reported that only preheating treatment of milk at >70 °C during commercial processing operations results in a number of physicochemical changes in the milk constituents, in particular, denaturation of whey proteins that interact with the synthetic fat globules [20,38,39].

“Milk flavor”, “butter” and “aroma” are several important indicators for evaluating sample preference. From the preference point of view, the samples with higher scores in “milk flavor”, “butter” and “aroma” are more popular. From a textural perspective, there were no significant differences between samples. This indicates that all samples were homogeneous in texture during the evaluation and there were no impurities noted when swallowing. In addition, viscosity is an important part of texture, and researchers have pointed out that the viscosity of skim milk does not show significant changes when the preheating temperature is below 60 °C [40]. Sensory results indicated that samples produced by a processing temperature of 50 °C and for a time for 30 min were preferred.

### 3.2. Principal Component Analysis and Cluster Analysis

The acquired e-tongue data was evaluated using principal components analysis (PCA). PCA was performed with means centering and Pareto scaling to account for the variation of peak intensities within the chromatograms [41]. This is a convenient statistical method and it can identify and express the data in such a way that highlights their differences and similarities. It reduces the amount of data to a smaller number of new derived variables which represent the original data adequately [42]. Classical 90% confidence ellipses were used in determining the sample populations in the scores plots. In the experiment of e-tongue analysis, except the CK, RM was taken for analyzing and comparing to explain fully variability among different processing samples and find out the optimal processing conditions which made the samples more perfected.

Cluster analysis (CA) is a generic method for a wide range of exploratory multivariate techniques. It is used for classifying a data set in homogeneous groups and represented in dendrogram which provides a simple way of visualizing the hierarchical structure of the clustering and the level at which each cluster is formed [43]. In order to diagnose and characterize the correlation among samples of different treatments, the resulting dendrogram was used for showing the similarity of the properties of e-tongue.

From Figure 3 and Figure 4, the main differences are found between samples processed with different temperature and for different times, respectively. Besides, CK presents the largest difference compared to samples of preheating as shown in Figure 3b, while in Figure 4b, the difference of CK is not direct. Based on the diversity of preheating temperature and preheating time on CK, it could be concluded that the effects of preheating temperature is significant compared to preheating time.

### 3.3. GC-MS

#### 3.3.1. Alkanes

Researchers have reported that alkanes are some of the most frequent compounds formed in milk when preheated [43]. Studies showed that alkanes like heptane, octane, decane, nonane, and undecane were produced in yoghurts made from cow, buffalo, ewe, and goat’s milks and heptane showed the highest concentration [44]. However, the concentration of alkane compounds is almost irrelevant to the flavor because of the high thresholds [43]. Alkanes could originate from forages. Tetradecane was the only alkane compound found in most skim milk samples and tetradecane has been reported in milk and cheese in previous studies [45,46]. In addition, researchers discovered a significant (*p* < 0.05) difference in tetradecane levels between cheeses made from milk obtained from farms located on the plain where cows grazed on pasture and milk obtained from farms located in the mountains where cows were fed hay [46].

#### 3.3.2. Acids

These important flavor components were found in higher concentration in skim milk samples. Fatty acids are produced primarily via lipolysis of milk fat [43]. The enzymes used come from the milk itself (lipoprotein lipase) and psychrotrophic bacteria growing in RM. Besides, fatty acids also originate from the degradation of lactose and amino acids, especially short chain fatty acids [47]. Short-chain and moderate-chain fatty acids have a significant impact on the aroma of dairy products because of their lower perception thresholds, especially butyric acid and hexanoic acid, for which the threshold value is lower than 0.3 mg/L [48]. Fatty acids contribute to the flavor of many dairy products, while at high quantities they cause hydrolytic rancidity [47]. Combined with sensory evaluation, which indicated the quantities of fatty acids was appropriate, the flavor of the sample preheated at 50 °C for 30 min was preferable. Furthermore, fatty acids are not only aroma compounds by themselves, but also serve as precursors of other compounds, such as methyl ketones, aldehydes, lactones, esters, and secondary alcohols [47]. Relative to the preheating treatment, ultrasound treatment significantly (*p* < 0.05) increased the content of fatty acids, especially hexanoic acid, octanoic acid and *n*-decanoic acid [49].

#### 3.3.3. Ketones

Ketones, being present mainly in the form of methyl ketones, are derivatives of free fatty acids that are first oxidized to *β*-ketoacids and then decarboxylated to the corresponding methyl ketones [47]. They have distinctive odors, such as fruity, floral, and musty notes contributing to the flavor of milk, and low perception thresholds. Very low concentration of ketone compounds were detected in skim milk samples. This result caused by the removal of fat in our skim milk production compared to the whole milk [43,50]. Especially 2-heptanone, a typical volatile compound in dairy products [51], was not detected in our skim milk samples. A similar result was reported [47] in strained yoghurt without fat and the report showed that ketones were decreased by reducing the fat content. Previous studies combined with this study indicated that ketones might one of the reasons why skim milk is inferior to whole milk.

#### 3.3.4. Aldehydes

Aldehydes are major secondary products of the autoxidation of unsaturated fatty acids where the primary products are hydroperoxides which undergo further degradation to hydrocarbons, alcohols and carbonyl compounds [52]. Meanwhile, aldehydes, intermediate and unstable compounds that are usually reduced to alcohols, appear at low concentrations in the volatile fraction of most skim milk samples and the results were similar to previous reports [53,54]. Fortunately, aldehydes are not typical compounds in milk and the low level of aldehydes indicated an optimal processing [51]. Low concentrations of aldehydes are characterized by green grass-like, herbaceous aromas and they contributes to the fresh flavor of milk, while at a high concentration they may cause off-flavors [51].

#### 3.3.5. Alcohols

Two alcohols (2-ethyl-1-hexanol, 1-octanol) were detected in all samples. Alcohols, which are responsible for the pleasant flavors in dairy products, could be produced by reduction from the aldehydes, and amino acid metabolism [50]. The presence of branched-chain primary alcohol indicates the reduction of the aldehyde produced from leucine [55]. Most volatile alcohols when at a slightly high concentration or unsaturated might have significant impact on food flavors because of their high odor threshold [50].

#### 3.3.6. Sulfur Compounds

Only one sulfur compound (dimethyl sulfone) was detected in the skim milk samples. Shibamoto et al. reported that dimethyl sulfone was also detected in milk heated at various temperatures (30–150 °C) without dimethyl sulfide being detected. In addition, the researchers explained that dimethyl sulfone is possibly formed as a result of dimethyl sulfide oxidation via dimethyl sulfoxide as the intermediate and it has also been found in fresh raw milk [56]. Reports have shown the presence of this compound in raw milk is influenced by the feed composition and this might explain why dimethyl sulfone was detected in CK [57,58,59].

## 4. Materials and Methods

### 4.1. Materials

Internal standard 2-methyl-3-heptanone and normal alkanes (C_6_–C_30_) were obtained from Sigma-Aldrich (Poole, Dorset, United Kingdom). The reference compounds used for identification were mainly obtained with purities over 95% (GC). Octanal (99%) and 1-octanol (99.5%) were supplied by Macklin Biochemical Co., Ltd. (Shanghai, China). Nonanal (96%) was purchased from Beijing Peking University Zotep Co., Ltd. (Beijing, China). 2-Nonanone (>98%), 2-undecanone (>98%), acetophenone (>95%), hexanoic acid (>98%), octanoic acid (>98%), *n*-decanoic acid (>98%), tetradecane (>99%) and 2-ethyl-1-hexanol (>99.5%) were obtained from TCI (Shanghai, China). Decanal (97%) and dimethyl sulfone (99%) were supplied by J&K Chemical Ltd. (Beijing, China).

Aqueous solutions of 0.1mol/L HCl used for calibrating, conditioning and cleaning of the e-tongue and 0.1 mol/L solutions of NaCl and monosodium glutamate (MSG) were supplied by Alpha M.O.S., (Toulouse, France) as a 1 mol/L concentrate which was diluted using distilled water immediately prior to use.

### 4.2. Experiment Design and Methods

#### 4.2.1. Raw Material Collection

Raw whole milk obtained from Beijing Sanyuan Food Corp., Ltd. (Beijing, China) was placed in the light resistant and refrigerated containers and then carried back to the laboratory within two hours. After that, the raw milk was kept at 4 ± 1 °C for 30 min until further preprocessing. The compositions of raw milk were analyzed by using a MilkoScan ^TM^ Minor instrument (Fossomatic, Foss Electric, Hillerød, Denmark). The composition was 3.12% (*w/w*) protein, 3.67% (*w/w*) lactose and 3.56% (*w/w*) fat.

#### 4.2.2. Samples Preparation

To compare differences between different preheating treatments, 11 groups of raw milk materials were prepared for various processing conditions. Four of the raw milk materials were severally preheated at 30 °C, 40 °C, 50 °C and 60 °C for 30 min prior to defatting and these samples were named as W30, W40, W50 and W60, respectively. Five of the materials were preheated at the optimal temperature for 10 min, 20 min, 30 min, 40 min and 50 min prior to defatting, respectively. These samples were tagged as T10, T20, T30, T40 and T50. One of the materials was divided to the control and not subjected to any preheating treatment before defatting. It was named as CK. The last of the raw milk materials was named RM and was not subjected to preheating or defatting. The preheated samples W30, W40, W50, W60, T10, T20, T30, T40, T50 and CK were centrifuged at 5000× *g* at 4 °C for 10 min. All experimental samples were homogenized at 200/50 Bar, and pasteurized at 70 °C for 30 min, then rapidly cooled to 4 °C. Then all samples were stored at 4 °C for further analysis.

#### 4.2.3. Sensory Analysis

Sensory analysis was performed by quantities descriptive analysis (QDA) [60]. The panelists consisted of Master’s degree students and laboratory technicians from Beijing Technology and Business University, and they were selected based on regular consumption of milk and time availability. A total of five panelists (four females, one male, aged 22–30) were screened and trained [61]. The sensory evaluation process was conducted in individual experiment table in the laboratory. A pencil, evaluation forms and paper napkin were supplied [62]. The panelists were provided with potable water and plain crackers (Pacific, Shanghai, China) for palate cleansing between samples. The descriptors (attributes) and definitions are shown in Table 4. Each sensory attribute was scored using the 9-point scales, 0 means no such sensory attribute feature, and 9 means sensory attribute feature is maximum. Before assessment, the samples were preheating at 35 °C to simulate oral temperature. Sensory evaluation was performed in duplicate (Interval 20 min).

#### 4.2.4. e-Tongue Analysis

An α-Astree Liquid Taste Analyzer (Alpha M.O.S., Toulouse, France) was used in this study. A taste sensor set (ZZ, JE, BB, CA, GA, HA and JB, all from Alpha M.O.S.), an Ag/AgCl reference electrode and a liquid and taste analyzer for data acquisition and auto-sampler control (Alpha M.O.S.). The e-Tongue was connected to a computer with Alpha M.O.S statistical software (Alpha M.O.S., Toulouse, France) installed. The sensors employed in this study were chemically modified field effect transistors (CHEMFETs), and specially designed by the manufacture for food and beverage analysis to ensure good sensitivity and cross-selectivity of each sensor. The electronic tongue was activated and calibration performed prior to use to make sure that the data collected was reliable and stable [63,64]. The samples were measured at room temperature and the test volume was 20 mL. The sensor array of the e-tongue was immersed in an aliquot of each sample for a period of 120 s. The average mV reading of every second of analysis was used for the statistical calculations. Each sample was analyzed 10 times, with the first three analyses were disregarded (as per the manufacturer’s instructions) due to varied or unstable mV readings [65].

#### 4.2.5. Headspace Extraction of Volatile Compounds

Volatile compounds were extracted from sample headspace using solid-phase microextraction (SPME) with a 1 cm fused silica PDMS/DVB fiber from Supelco (Bellefonte, PA, USA). A sample (8 mL) of the skim milk was added to a standard 20 mL headspace vial (equipped with a cap with a polytetrafluoroethylene septum) and 10 μL of 6.6 mg/L 2-methyl-3-heptanone in *n*-hexane was added as internal standard [41]. The vial was immersed in a 50 °C water bath and stirred by a magnetic stirrer to stabilize for 20 min. The SPME fiber was exposed to the sample headspace and extracted for 30 min.

#### 4.2.6. GC-MS Analysis

After extraction, the volatiles were desorbed in the splitless injector of a GC-MS system (7890A-5975C, Agilent Technologies, Santa Clara, CA, USA) for 5 min. A DB-WAX capillary column (30 m × 0.32 mm × 0.25 μm, Agilent Technologies, Inc., Palo Alto, CA, USA) was used. Helium was used as a carrier gas at a constant linear gas flow velocity of 1.0 mL/min [66]. The injector temperature was 250 °C. Splitless mode was used with a 5 min desorption time. The initial oven temperature was 35 °C for 1 min, followed by a rate of 5 °C/min to 200 °C held for 5 min (total run time 39 min). The MS transfer line temperature was 230 °C. MS spectra in election ionization mode (MS-EI) were recorded with a 70 eV ionization energy, and the ion source temperature was set at 230 °C. Full-scan acquisition was used in the 30–350 amu range, and solvent delay was set as 5 min.

#### 4.2.7. Qualitation and Semi-Quantitation

##### Qualitation of Volatile Compounds

Compounds were identified based on retention indices, mass spectral library and authentic standard compound comparisons. Mass spectrometry was performed by comparing the mass spectra of unknown peaks with those stored in the National Institute of Standards and Technology, (NIST, Gaithersburg, MD, USA) library [67]. Kováts retention indices were determined by injection of a solution containing the homologous series of normal alkanes (C_6_-C_30_) in a temperature-programmed run [68], as described above, and these values were compared with those reported in http://webbook.nist.gov/chemistry/cas-ser.html. In addition, the main volatiles in milk were confirmed by comparing the retention times of gas chromatographic peaks to those of authentic standard compounds. A compound was considered to be unambiguously identified if it had matching retention indices with the reference compound or literature data and additionally matching mass spectra.

##### Quantitation of Volatile Compounds

Identified volatile compounds were subjected to semiquantitative analysis based on comparison of HS-SPME peak areas of analyzed compounds with that of internal standard (2-methyl-3-heptanone).

#### 4.2.8. Statistical Analysis

PCA models were created by The Unscrambler X version 10.4 (CAMO Software As., Oslo, Norway) and Cluster analysis (CA) was conducted by SPSS 23 (IBM Deutschland GmbH, Ehningen, Germany) in order to identify the optimal conditions for skim milk preprocessing. Data were expressed as mean ± SD. All statistical analyses were processed with one way analysis of variance (ANOVA) using SPSS 23. The criterion for statistical significance in all tests was *p* < 0.05. GraphPad Prism 7 (GraphPad Software, Inc., La Jolla, CA, USA) and OriginPro 2017 (OriginLab Corporation, Northampton, MA, USA) were performed to all the figures.

## 5. Conclusions

Comparison of skim milk samples with different preheat processing conditions were analyzed by sensory evaluation, e-tongue and SPME-GC-MS. Sensory evaluation results showed that samples W50 and T30 displayed excellent scores and were the most preferred in overall feel, respectively. E-tongue analysis results were associated using PCA and CA. What can be observed from the cluster formations using PCA and CA is that the e-tongue can distinguish the skim milks clearly. W50 and T30 were classified as the same cluster with RM, which indicates they have the highest similarity in taste responses measured by e-tongue sensors. The qualitative analysis of skim milk volatiles using GC-MS is a common way and the 13 major volatile compounds were analyzed. Obviously, compared to previous studies of whole milk, most volatile compounds were decreased by decreasing the fat content, especially ketones and the typical volatile compound 2-heptanone was not detected in samples. W50 and T30 had the highest concentrations of acids contributing to the milk flavor of samples. As for alcohols, there was no significant difference between samples. In conclusion, combining the results of sensory, e-tongue and GC-MS, preheat treatments is an effective way to upgrade the quality of skim milk compared to CK. Furthermore, compared to other preheating treatments in this study, preheating at 50 °C for 30 min before defatting is conducive to significantly improved skim milk quality.

## Figures and Tables

**Table 1:** Advantages and disadvantages of various milk processing methods.

Processing Methods	Advantages	Disadvantages
Thermal treatment	Provide acceptable safety and shelf life, improve the functional properties [20]	May give rise to chemical and physical changes and reduce the content or bioavailability of some nutrients when ultra-high temperature [28]
Ultrasonic treatment	Homogenization of milk due to smaller fat globules with a granular surface [28]	Difficult to become a commercial process on its own [21]
High pressure	Minimal effects on the sensory and nutritional quality and as a tool for modification of macromolecular constituents [20]	Require a large capital investment [29]

**Table 2:** Volatile compounds identified by GC-MS in skim milks with different preheating temperatures.

No.	Compounds	RT/min ^d^	RI		Concentration (μg/L)					Identification ^h^
			Cal. ^e^	Ref. ^f^	CK	W30	W40	W50	W60	
**Ketones**										
1	2-Nonanone	14.47	1310	1366	0.57 ± 0.11	0.50 ± 0.23	0.40 ± 0.07	0.47 ± 0.10	0.69 ± 0.08	MS/STD/RI
2	2-Undecanone	19.73	1509	1599	0.41 ± 0.06	0.53 ± 0.10	0.66 ± 0.11	0.43 ± 0.32	0.21 ± 0.14	MS/STD/RI
3	Acetophenone	20.87	1555	1627	0.06 ± 0.01	0.03 ± 0.00	0.04 ± 0.01	0.07 ± 0.03	0.05 ± 0.02	MS/STD/RI
Total					1.05 ± 0.12	1.06 ± 0.24	1.10 ± 0.16	0.97 ± 0.39	0.96 ± 0.22	
**Acids**										
1	Hexanoic acid	25.74	1765	1850	1.03 ± 0.33	0.47 ± 0.35	0.54 ± 0.11	1.33 ± 0.01	- ^g^	MS/STD/RI
2	Octanoic acid	30.06	1969	2038	7.55 ± 2.53	6.48 ± 2.16	6.04 ± 0.36	14.75 ± 7.97	4.48 ± 2.88	MS/STD/RI
3	n-Decanoic acid	34.07	2176	2246	6.60 ± 2.06	4.80 ± 0.85	3.88 ± 1.13	9.94 ± 4.92	1.59 ± 0.64	MS/STD/RI
Total					15.18 ± 3.50 ^ab^	11.75 ± 1.92 ^b^	10.46 ± 0.78 ^b^	26.01 ± 12.76 ^a^	6.06 ± 3.48 ^b^	
**Aldehydes**										
1	Octanal	11.78	1214	1292	-	-	-	0.18 ± 0.14	-	MS/STD/RI
2	Nonanal	14.55	1313	1390	0.56 ± 0.18	0.43 ± 0.07	0.46 ± 0.17	1.42 ± 0.88	0.44 ± 0.11	MS/STD/RI
3	Decanal	17.23	1412	1472	-	1.27 ± 0.81	0.18 ± 0.09	0.98 ± 0.37	1.21 ± 0.73	MS/STD/RI
Total					0.56 ± 0.18 ^b^	1.70 ± 0.79 ^ab^	0.64 ± 0.25 ^b^	2.58 ± 1.17 ^a^	1.65 ± 0.61 ^ab^	
**Alcohols**										
1	2-Ethyl-1-hexanol	17.16	1410	1484	3.93 ± 0.20	2.33 ± 0.73	3.86 ± 1.78	4.23 ± 3.19	2.16 ± 0.99	MS/STD/RI
2	1-Octanol	18.83	1474	1558	0.27 ± 0.04	0.24 ± 0.07	0.20 ± 0.10	0.51 ± 0.33	0.10 ± 0.02	MS/STD/RI
Total					4.20 ± 0.17	2.58 ± 0.80	4.06 ± 1.88	4.73 ± 3.51	2.26 ± 1.01	
**Alkanes**										
1	Tetradecane	14.81	1323	1400	-	0.11 ± 0.00	-	0.15 ± 0.01	0.17 ± 0.01	MS/STD/RI
Total					-	0.11 ± 0.00^c^	-	0.15 ± 0.01^b^	0.17 ± 0.01^a^	
**Sulfur compounds**										
1	Dimethyl sulfone	26.28	1789	1895	0.10 ± 0.00	-	-	0.15 ± 0.10	-	MS/STD
Total					0.10 ± 0.00	-	-	0.15 ± 0.10	-	

^a, b, c^ Significant (*p* < 0.05) difference between samples. ^d^ Retention time on DB-WAX column. ^e^ Retention indices calculated on DB-WAX column against n-alkanes. ^f^ Retention indices reported by http://webbook.nist.gov/chemistry/cas-ser.html. ^g^ not detected. ^h^ Volatiles were identified according to abbreviations: MS, comparing mass spectrum with those in NIST library. STD, comparing the retention time of compounds in samples to authentic standards. RI, comparing retention indices (RI) on DB-Wax column with those in the literature.

**Table 3:** Volatile compounds in the skim milks of different preheat time identified by GC-MS.

No.	Compounds	RT/min ^c^	RI		Concentration (μg/L)						Identification ^g^
			Cal. ^d^	Ref. ^e^	CK	T10	T20	T30	T40	T50	
**Ketones**											
1	2-Nonanone	14.47	1310	1366	0.57 ± 0.11	0.80 ± 0.01	1.15 ± 0.59	0.47 ± 0.10	0.90 ± 0.02	1.05 ± 0.13	MS/STD/RI
2	2-Undecanone	19.73	1509	1599	0.41 ± 0.06	- ^f^	0.90 ± 0.22	0.43 ± 0.32	0.44 ± 0.12	0.46 ± 0.14	MS/STD/RI
3	Acetophenone	20.87	1555	1627	0.06 ± 0.01	-	0.09 ± 0.01	0.07 ± 0.03	0.03 ± 0.00	0.05 ± 0.02	MS/STD/RI
Total					1.05 ± 0.12 ^b^	0.80 ± 0.01 ^b^	2.15 ± 0.82 ^a^	0.97 ± 0.39 ^b^	1.38 ± 0.13 ^ab^	1.55 ± 0.28 ^ab^	
**Acids**											
1	Hexanoic acid	25.74	1765	1850	1.03 ± 0.33	-	-	1.33 ± 0.01	-	-	MS/STD/RI
2	Octanoic acid	30.06	1969	2038	7.55 ± 2.53	4.06 ± 2.66	7.13 ± 2.52	14.75 ± 7.97	9.19 ± 3.14	9.97 ± 2.16	MS/STD/RI
3	n-Decanoic acid	34.07	2176	2246	6.60 ± 2.06	3.50 ± 1.98	6.26 ± 3.04	9.94 ± 4.92	7.07 ± 2.12	6.90 ± 3.69	MS/STD/RI
Total					15.18 ± 3.50 ^ab^	7.56 ± 4.64 ^b^	13.39 ± 5.35 ^ab^	26.01 ± 12.76 ^a^	16.26 ± 5.16 ^ab^	16.87 ± 5.78 ^ab^	
**Aldehydes**											
1	Octanal	11.78	1214	1292	-	-	-	0.18 ± 0.14	-	0.23 ± 0.09	MS/STD/RI
2	Nonanal	14.55	1313	1390	0.56 ± 0.18	0.81 ± 0.26	1.41 ± 0.63	1.42 ± 0.88	0.59 ± 0.15	1.46 ± 0.33	MS/STD/RI
3	Decanal	17.23	1412	1472	-	1.46 ± 0.29	1.86 ± 0.63	0.98 ± 0.37	2.33 ± 1.55	4.39 ± 2.13	MS/STD/RI
Total					0.56 ± 0.18 ^b^	2.27 ± 0.03 ^b^	3.27 ± 1.24 ^b^	2.58 ± 1.17 ^b^	2.91 ± 1.70 ^b^	6.09 ± 2.54 ^a^	
**Alcohols**											
1	2-Ethyl-1-hexanol	17.16	1410	1484	3.93 ± 0.20	3.63 ± 0.34	5.92 ± 3.06	4.23 ± 3.19	2.46 ± 0.14	2.17 ± 0.69	MS/STD/RI
2	1-Octanol	18.83	1474	1558	0.27 ± 0.04	0.19 ± 0.03	0.40 ± 0.10	0.51 ± 0.33	0.20 ± 0.01	0.47 ± 0.18	MS/STD/RI
Total					4.20 ± 0.17	3.82 ± 0.37	6.32 ± 3.14	4.73 ± 3.51	2.65 ± 0.15	2.64 ± 0.87	
**Alkanes**											
1	Tetradecane	14.81	1323	1400	-	0.34 ± 0.17	0.93 ± 0.13	0.15 ± 0.01	0.19 ± 0.05	0.14 ± 0.05	MS/STD/RI
Total					-	0.34 ± 0.17 ^b^	0.93 ± 0.13 ^a^	0.15 ± 0.01 ^b^	0.19 ± 0.05 ^b^	0.14 ± 0.05 ^b^	
**Sulfur compounds**											
1	Dimethyl sulfone	26.28	1789	1895	0.10 ± 0.00	-	-	0.15 ± 0.10	-	-	MS/STD
Total					0.10 ± 0.00	-	-	0.15 ± 0.10	-	-	

^a, b,^ Significant (*p* < 0.05) difference between samples. ^c^ Retention time on DB-WAX column. ^d^ Retention indices calculated on DB-WAX column against n-alkanes. ^e^ Retention indices reported by http://webbook.nist.gov/chemistry/cas-ser.html. ^f^ not detected. ^g^ Volatiles were identified according to abbreviations: MS, comparing mass spectrum with those in NIST library. STD, comparing the retention time of compounds in samples to authentic standards. RI, comparing linear retention indices (RI) on DB-Wax column with those in the literature.

**Table 4:** Flavor attributes selected of sensory evaluation.

No.	Sensory Attributes	Evaluation Method
1	Aftertaste (milk flavor)	After tasting, intensity of residual milk flavor.
2	Aftertaste (sweetness)	After tasting, intensity of residual sweetness.
3	Aftertaste (fat-sense)	After tasting, intensity of fat feeling in the mouth.
4	Aroma	Inherent flavor of milk, the aroma is gentle, scented, natural and without off-flavor.
5	Sweetness	Intensity of the inherent sweetness for sample.
6	Off-flavor	Smell should not appear in milk, such as stink and musty.
7	Sourness	Intensity of sourness was felt in the mouth.
8	Saltiness	Intensity of salt were felt in the mouth. Slight salt should exist in the optimum sample
9	Milk flavor	Intensity of inherent milk flavor was felt in the mouth.
10	Butter	Intensity of greasy feeling in the mouth.
11	Texture	Whether the sample is a uniform liquid, and whether there is a clot or precipitation.
